# Knowledge of school teachers and the associating factors in the effective delivery of the school health programme in Nigeria

**DOI:** 10.11604/pamj.2022.43.4.30838

**Published:** 2022-09-02

**Authors:** Ayoola Oluwaseun Bosede, Taiwo Akinyode Obembe, Ayodeji Matthew Adebayo

**Affiliations:** 1 Department of Environmental Health Science, School of Health Technology, Federal University of Technology, Owerri, Nigeria,; 2 Department of Health Policy and Management, Faculty of Public Health, College of Medicine, University of Ibadan, Ibadan, Nigeria,; 3 Department of Community Medicine, College of Medicine, University of Ibadan, Ibadan, Nigeria

**Keywords:** School health programme, school health services, school community, school-age children, knowledge level, school teacher, role perception, awareness level

## Abstract

**Introduction:**

the important position that teachers occupy in school settings make them indispensable in the effective delivery of the School Health Programme (SHP). This study assessed the SHP knowledge of primary school teachers and the perception of their roles in the successful delivery of the programme in Ondo State, Nigeria.

**Methods:**

this was a cross-sectional descriptive study. A multi-stage sampling technique was used to select 400 teachers from 42 primary schools, from the study population. A semi-structured self-administered questionnaire was used to collect data. Each respondent´s aggregate score was classified as being poor knowledge, if it was < 50% of the maximum obtainable score and good knowledge, if it was equal or more than 50% of the maximum obtainable score.

**Results:**

the majority of the teachers (76%) demonstrated poor knowledge of the programme. Yet, a good number of them, believe they have a part to play in the implementation of the SHP. Academic qualification was found to be statistically associated with SHP knowledge (p < 0.01). Length of time in teaching service (p= 0.035; OR=1.033; 95%CI = 1.002- 1.065) was found to be a predictor of adequate SHP knowledge.

**Conclusion:**

the SHP knowledge of the teachers was found to be inadequate. Although, most of the teachers agreed that they had roles to play in the SHP delivery, a sizeable number of them could not state what those roles entailed. It is recommended, therefore, that government and all stakeholders in education and health sectors should conduct trainings on SHP, focusing on teachers´ roles in the school community.

## Introduction

School Health Programme (SHP) is a health programme directed to meet the health needs of students and laying a good foundation for their future, with the support of the home, community, and government [1]. It is defined as the totality of projects and activities in a school environment, which are designed to protect and promote the health and development of the school community [2]. The objectives of the SHP are to obtain rapid and sustained improvement in the health of school children, to ensure that children from preschool age to adolescence are in optimum health at all times so that they can attain their physical and intellectual potential, as well as to receive maximal moral and emotional benefits from health providers, teachers, and the school environment [2]. The advantages of effective school health services cannot be over-emphasized. When effectively managed, school health services have yielded significant contributions in school based health programmes [3]: health related interventions [4], feeding [5], smoking cessation programmes [6], in primary prevention of cardiovascular diseases in children [7] and in detecting large citywide epidemics [8]. Besides augmenting care for the populace, research indicates that effective school health programmes help to increase school attendance and academic performance, as well as decrease school drop-out rates [9]. Therefore, the inter-related linkage between proper oral/school health, academic performance and other long-term outcomes is notable.

The SHP in practice involves government, education and health professionals. The programme is described as the cooperative activities of school teachers, physicians, dentists and others so as to appraise, promote, protect and maintain the health of all pupils and the school personnel [10]. The bulk of the SHP service revolves around the teachers, who explain to the pupils and their parents, the health appraisal findings. They also help in the early identification of diseases and impairments, thereby helping to prevent and control diseases. In addition, they provide emergency services for sudden illnesses and injuries. He/she is in a good position to use personal examples in lifestyle habits and experiences to health educate the children [10]. Thus, teachers are in a vantage position to promote positive health and wellbeing of school age children through the SHP. The lack of knowledge of the functioning of the programme and their roles in the programme delivery will, therefore, impede its effective implementation [10]. A sound and effective health education, emphasizing knowledge, attitudes and practices to teachers during trainings provides the foundation for successful implementation of the SHP [11], hence, the need for teachers to be knowledgeable in the functioning of the SHP. Various studies have been conducted since the release of the National School Health Policy (NSHPo) by the Nigeria Federal Ministry of Education (FME). Most of these studies have reported inadequate knowledge among the assessed teachers [10,12,13]. This unfavorable trend has continually reflected on the Quality of Implementation (QoI) of the programme in schools, with many researchers reporting poor to below average QoI of SHP [14-16]. These studies on the SHP knowledge of the school teachers are needed to improve on the current suboptimal quality of implementation of the SHP in Nigeria. This study was therefore, conducted to assess the SHP knowledge of primary school teachers in Ondo State. The findings from this study may inform further training and retraining needs of teachers for improved SHP delivery in the State and the country at large.

## Methods

**Study design:** a descriptive cross-sectional study design was used; the study covered two state districts - urban and rural.

**Study population:** the two districts were purposively chosen, with the urban one being the state capital, providing a true representation of the situation in the state. The rural district was chosen for its proximity to the urban district. This research is part of a larger study on the appraisal of the SHP in the Ondo State, and the data were collected between August 2019 and December 2019. A minimum sample size of 223 respondents was determined using the Leslie Kish formula for determining single proportion for descriptive studies [17]. Considering that prevalence “P” of 15.4% represents the proportion of teachers with adequate SHP knowledge [10], a total of 400 school teachers which were seeing in the schools at the time of data collection and which gave their written informed consents were selected from 42 schools which were already enrolled into the study through simple random sampling technique.

**Data collection method:** a semi-structured self-administered questionnaire, containing a 35-point knowledge item [13] and an eight point role perception item [12], was used to test the SHP knowledge of the school teachers and their perception regarding the implementation of SHP. There were 5 close-ended questions with 20 maximum obtainable points and 5 open-ended questions with 15 maximum obtainable points on teachers’ knowledge of the SHP. Each correct option was scored 1 mark for the close-ended question and 3 marks for the open-ended questions, and each wrong answer was scored zero; giving minimum and maximum obtainable scores of 0 and 35, respectively. The principle of negative marking was applied where the total mark obtained were deducted from the total mark that should have been scored, if wrong answers were not selected. This was to check respondents selecting options “Yes”, “No” and “I don´t know” randomly. Each respondent´s aggregate score was classified as being inadequate (poor) knowledge if it was < 50% of the maximum obtainable score (< 17.5) and adequate (good) knowledge if it was equal or more than 50% of the maximum obtainable scores (≥17.5) [13].

**Data analysis:** the data from the questionnaires were entered into the computer and analyzed using the Statistical Package for Social Sciences version 21.0 [18]. Frequencies and percentages were estimated for categorical variables (gender, age categories, religion, ethnicity, knowledge of SHP) while summary indices such as means and standard deviations were generated for continuous variables (length of time in teaching service, age). Inferential statistics (Chi-square test) was used to test for associations between selected categorical variables. Logistic regression was also employed to determine the predictor variables of SHP knowledge. Instruments with grossly missing data were expunged from the analysis.

**Reliability of the data collection instrument:** reliability test was carried out to assess the accuracy of the data collection instruments. A pre-test was carried out in two selected schools in the State, one from an urban district and the other from a rural district, other than the selected districts for the research. Reliability of the instruments were ascertained using a Cronbach´s alpha test (with a score of 0.721) that was carried out on the data collected during the pre-test.

### Ethical approval and consent to participate

Ethical clearance for this study was obtained from the Ondo State Health Research Ethics committee (OSHREC/01/07/19/137). Details of the study were explained to the school heads and teachers. All participants gave their written informed consent.

## Results

Four hundred questionnaires were distributed, out of which 24 were either irretrievable or not properly completed. Thus, a total of 376 questionnaires were analyzed for the study (a response rate of 94%). Overall, the rating of respondents´ knowledge of SHP revealed that the majority (76.9%) of the teachers had inadequate knowledge of SHP. Table 1 shows socio-demographic characteristics of the respondents. Overall, the mean age of teachers was 36.3 ± 9.4 years with the higher proportion of respondents in the age group 26 - 35 years. Overall, the mean length of time in teaching service was 7.7± 7.5 years, with the majority in the range 10 years and below. Majority of the respondents were female (79.8%). Educational records of the respondents revealed that the lowest educational attainment was a diploma in education (4.8%) while the highest were postgraduate degrees (3.2%). Majority of the respondents had National Certificate in Education (44.1%). Findings also showed that 93.9% of the respondents were Christians, 5.6% were Muslims and only one respondent belonged to the African Traditional Religion (ATR). Most of the respondents representing 90.2% of the total were of the Yoruba ethnic group, 4% were Igbos, and the remaining 5.9% were from other ethnic groups in Nigeria.

**Table 1:** socio-demographic characteristics of respondents

Respondent´s age (Years)	Frequency N= 376	Percentage (%)
16-25	43	11.4
26-35	164	43.6
36-45	94	25.0
46-55	62	16.5
56-60	13	3.5
Mean age	**36.3 ± 9.4**	
**Length of time in teaching (Years)**		
≤10	270	71.8
11-20	77	20.5
21-30	28	7.4
≥31	1	0.3
Mean	**7.7 ± 7.5**	
Gender		
Male	76	20.2
Female	300	79.8
**Education**		
Diploma in education	18	4.8
National certificate in education	166	44.1
Higher National Diploma	24	6.4
University degree	156	41.5
Masters/PhD	12	3.2
**Religion**		
Christianity	353	93.9
Islam	21	5.6
African Traditional religion	1	.3
**Ethnicity**		
Yoruba	339	90.2
Igbo	15	4.0
Others	22	5.9

Table 2 shows the awareness status of the respondents on the National School Health Policy (NSHPo). When asked if they have ever heard of the NSHPo, 63.6% of the respondents had never heard of the NSHPo. On the question that sought to find out whether or not the respondents had seen the NSHPo document, 89.6% of respondents had never seen the policy document before. On whether or not the respondents have had previous training on the School Health Programme using the national SHP implementation guidelines, only 7.7% of the respondents answered in the affirmative. Table 2 also shows the respondents´ perception of their roles in the SHP implementation. From the table, majority of the respondents (77.4%) believed that they have roles to play in the SHP implementation. When asked to list four of their roles in SHP implementation, a majority (73.4%) of the respondents could not mention a single role. Only 1.6% of the respondents listed four correct roles that are expected of them in SHP implementation.

**Table 2:** awareness of the National school health policy and SHP role perception

Ever heard of the National School Health Policy?	Freq N=376	Percentage (%)
Yes	137	36.4
No	239	63.6
**Ever seen the National School Health Policy Document?**	**Freq N=376**	**Percentage (%)**
Yes	39	10.4
No	337	89.6
**Ever had training on School Health Programme?**	**Freq N=376**	**Percentage (%)**
Yes	29	7.7
No	347	92.3
**Do you think that you have role(s) to play in SHP implementation?**	**Freq N=376**	**Percentage (%)**
Yes	291	77.4
No	85	22.6
**List four roles that are expected of you in the implementation of SHP**	**Freq N=376**	**Percentage (%)**
no role	276	73.4
1 role	25	6.6
2 roles	34	9.0
3 roles	35	9.3
4 roles	6	1.6

Table 3 shows the association between respondents´ socio-demographic characteristics and overall knowledge of SHP. The information on the tables as regards association between level of education and SHP knowledge shows a rise in level of knowledge with a commensurate increase in the level of education. The level of knowledge dropped with lower levels of education. The result shows a very strong statistical association between education of the respondents and their SHP knowledge level at p< 0.001. Respondents in the range of 50 - 60 years had the highest number of respondents with a good knowledge of SHP (38.5%). This is followed by respondents in the range of 46 - 55 years (24.6%). Respondents, in the age range 16-25 years, have the least proportion of respondents with good SHP knowledge (16.1%). This result was however not statistically significant (p = 0.521). In the association between the length of time in teaching service and the respondents´ SHP knowledge, it was discovered that respondents who had spent between 21-30 years in service exhibited more knowledge compared with those in other categories. Association between respondents´ teaching time and their SHP knowledge is however, not statistically significant (p= 0.162). Males have more respondents with adequate SHP knowledge (25.0%) when compared with their female counterparts (22.7%). The finding is also not statistically significant at p= 0.667.

**Table 3:** respondents’ socio-demographic characteristics and knowledge of SHP

Educational level of respondents	Knowledge level of the respondents as regards SHP		Total	X2	p-value
	Good	Poor			
Diploma in education	1 (5.6%)	17 (94.4%)	18	21.354	0.000*
National certificate in education	31 (18.7%)	135 (81.3%)	166		
Higher National Diploma	0 (0%)	24 (100%)	24		
University degree	52 (33.3%)	104 (66.7%)	156		
Masters/PhD	3 (25%)	9 (75%)	12		
**Respondents´ age**					
16-25	7 (16.3%)	36 (83.7%)	43	3.223	0.521
26-35	40 (24.2%)	125 (75.8%)	165		
36-45	20 (21.3%)	74 (78.7%)	94		
46-55	15 (24.6%)	46 (75.4%)	61		
56-60	5 (38.5%)	8 (61.5%)	13		
**Respondents´ length of time in teaching service**					
≤ 10	57 (21.1%)	213 (78.9%)	270	5.132	0.162
11-20	19 (24.7)	58 (75.3%)	77		
21-30	11 (39.3%)	17 (60.7%)	28		
≥31	0 (0.0%)	1 (0.3%)	1		
**Respondents´ gender**					
Male	19 (25%)	57 (75%)	76	0.186	0.667
Female	68 (22.7%)	232 (77.3%)	300		

* Significant value

Table 4 shows the predictors of overall knowledge of SHP. The model included age, length of time in teaching, qualification and gender. After adjusting for confounders, only length of time in teaching service and qualification of the respondents were found to be significant predictors of adequate knowledge of SHP. With an additional year spent in teaching service, there is likelihood of improved SHP knowledge (p= 0.035, OR=1.033; 95%CI = 1.002- 1.065). The analysis also shows that teachers with diploma certificates were less likely to have adequate SHP knowledge when compared with teachers with first degrees (p= 0.04, OR=0.118; 95%CI = 0.015- 0.908). Teachers with NCE were also less likely to have adequate SHP knowledge compared with teachers with first degrees (p= 0.003, OR=0.459; 95%CI = 0.275- 0.767).

**Table 4:** predictors of knowledge of SHP among the school teachers

Variables	Odds ratio	p-value	95% confidence interval
Age	1.016	0.215	0.991 - 1.042
Length of time in teaching service	1.033	0.035‡	1.002 - 1.065
**Qualification**			
*B. Sc/B.A/B.Tech	1.000		
Diploma	0.118	0.040‡	0.015 - 0.908
NCE	0.459	0.003‡	0.275 - 0.767
HND	0.000	0.998	0.000-
Masters/PhD	0.667	0.556	0.173 - 2.567
**Gender**			
*Male	1.000		
Female	0.879	0.667	0.490 - 1.579

* Reference variable, ‡significant value

## Discussion

This study was conducted to assess the knowledge of SHP among primary school teachers and the associating factors in the effective delivery of SHP in Ondo State, Nigeria. Overall, we found out that majority of the teachers had poor/inadequate knowledge of the programme. A good number believes that they are important to the SHP implementation, although they do not know what their roles are in SHP implementation.

The socio-demographic distribution revealed that most of the respondents were in the range 26- 35 years, with a majority hailing from the Yoruba ethnic group. Christians were also in the majority (Table 1). This finding on age is not consistent with past studies and a recently conducted study by Obembe *et al*. (2016), where most of the respondents were within the range of 40 - 49 years [13]. A similar study, by Adebayo *et al*., indicates the same age range of 40 – 49 years [10]. This difference may be associated with the fact that the current study includes respondents from both public and private schools, unlike the earlier cited studies, which were conducted in public schools alone. Private schools are known for employing young teachers, fresh graduates and secondary school-leavers, whereas government rarely employs new teachers in public schools. This explains the advanced age of respondents in public schools. The majority of female respondents in the current study are in tandem with the finding of Adebayo and Onadeko, and Ofovwe and Ofili [10,19]. Obembe *et al*., on the contrary, interviewed a majority male population [13]. This situation further confirms that there are more male teachers in secondary schools and more female teachers in the primary schools. Having more Yoruba ethnic group and Christian respondents, is in line with the two previously cited studies. This situation may be associated with the fact that Southwestern Nigeria, where the research was conducted, is a Yoruba and Christian dominated region of the country. The majority NCE holder population was also observed in the report of Adebayo and Onadeko, and Ofovwe and Ofili [10,19]. Obembe, in a 2016 study, reported that majority of the respondents were first degree holders [13]. This difference may be associated with the fact that Obembe *et al*., 2016 study was conducted among the secondary school teachers that usually have a large population of university and postgraduate degree holders.

The teachers were asked if they have ever heard of, seen or had any previous trainings on the NSHPo. The research showed that majority of the teachers have never heard of, seen or had any previous training on the NSHPo (Table 2). It is thus, safe to say that awareness level of the NSHPo was low among the teachers in primary schools in Ondo State. This finding about low levels of awareness is supported by the finding of Ademokun *et al*., in research that was carried out in Southwestern Nigeria in 2014, when it reported that many of the school head teachers had never heard of the 2006 NSHPo. The few who were aware, heard about it through the mass media. None of these people had neither seen nor read the policy document [20]. Obembe *et al*., (2016) in a study carried out among 426 school teachers in Ibadan in support of this finding, discovered that about one-third of the respondents had heard of NSHPo. A few of them, however, had seen the document [13]. Level of awareness was found to be high among the head teachers as reported by Ofovwe and Ofili in a research carried out among head teachers of primary schools in Edo State, where over 70% of the respondents claimed that they have heard about the NSHPo [19]. The differing reports are likely due to the fact that the 2007 Ofovwe and Ofili research, made use of only one variable ‘heard of´ to arrive at the level of awareness unlike this study, which considered other variables including “see” on and “previous training” to rate level of awareness. The poor/inadequate knowledge of SHP among the teachers as identified by our study may not be unconnected to the reported low level of awareness among these teachers, since awareness can help improve knowledge.

Our finding about poor knowledge of SHP among the teachers is in line with the report of Abodunrin *et al*. where it was discovered that only 22% of all the respondents have good knowledge of school health services, with more than half (51%) of the total respondents demonstrating very poor knowledge of School Health Services [21]. In another rural-urban comparative study conducted among primary school teachers in Oyo State Nigeria, it was discovered that 84.6% of the teachers had inadequate knowledge of SHP, with similar proportion in rural (84.2%) and urban (84.9%) schools [10], further corroborating the findings of this study. The report from Ofovwe and Ofili (2007) also supports this finding where it was reported that none of the 133 head teachers had adequate knowledge of SHP. The proportion of head teachers with poor knowledge of SHP from private schools, at 93.1%, was significantly higher than the proportion from public schools [19]. Obembe *et al*., 2016 however, reported a contrary finding when it was discovered that 55.9% of the total respondents demonstrated good knowledge of the SHP [13]. This difference may be as a result of the inherent differences in the construction of the instrument for data collection for both researches. It may also be as a result of the differences in the scoring system employed by this research.

Academic qualification of the school teachers was found to be significantly associated with the SHP knowledge (p < 0.01). It shows that teachers with university degrees have better knowledge of the SHP (Table 4). Results of the logistic regression model further corroborate the finding that academic qualification is a predictor of SHP knowledge. This is consistent with the finding of Obembe *et al*. [13] and Adebayo and Onadeko [10]. As the number of years in teaching service increased, there seemed to be improvement in SHP knowledge (Table 4). This finding though not statistically significant (p= 1.62) nevertheless was in consistence with establishing that length of time in teaching is a predictor of knowledge (p= 0.035, OR=1.033; 95%CI = 1.002- 1.065). While *Obembe et al*. employed age and educational qualification as the independent variables to predict SHP knowledge of the teachers [13], and Adebayo and Onadeko, 2016 used location of the school, age, marital status and numbers of qualification as variables to predict the SHP knowledge of the teachers [10], the findings from the two research are in line with the finding of this research at the points of intersection.

Majority of the teachers perceived that they have roles to play in the successful implementation of the SHP. When the teachers were asked to list some of their roles in the SHP implementation, the majority of them could not even mention one (Table 3). This further buttressed the previous submission that the majority of the teachers have inadequate knowledge of the SHP. Many other reports on teachers´ perception both locally and internationally have usually focused on what teachers think about the SHP as a whole, and not about their own roles in the successful implementation of the programme [22,23]. This research finding on role perception is consistent with the report of Adebayo and Onadeko in a research report of 2015, where it was reported that more than half of the teachers had poor perception of their roles in SHP. The same study revealed that majority of the teachers felt they had roles to play in the administration of SHP. In asking them to list four roles which were expected of them in the administration of the SHP, less than half of the teachers were able to list any of the four roles that are expected of them, only a few were able to list the four correctly [12]. This research aspect on role perception is intended to build up on the 2015 findings of Adebayo and Onadeko. This finding points out the fact that teachers are increasingly becoming aware of the fact that they essential to the implementation, sustenance and success of the SHP. Despite this, teachers still need to be trained to enhance the understanding of their roles in the implementation of SHP.

**Limitations:** this study is limited by the fact that it was conducted among the primary school teachers only, these findings, therefore, may not accurately describe the situations among the secondary school teachers in the state.

## Conclusion

Teachers are strategically placed to ensure effective, efficient and sustainable delivery of the SHP. It is, thus, very crucial for them to be well-informed about the programme, how it functions and what their roles should be in the delivery of the programme for the benefit of the school children and the entire school community. It is therefore recommended to the state government and all stakeholders in the school system should, as a matter of urgency, conduct SHP training for all the teachers in the state. Teachers should also be encouraged to develop themselves, as higher education has been found to be significantly related to knowledge.

**Funding:** no funding was received for this research.

### What is known about this topic

- *School teachers are important to the implementation of the School Health Programme;*
- *Awareness of the National School Health Policy is poor among the teachers;*
- *Most of the teachers believe that they have important roles in the implementation of the SHP*.

### What this study adds

- *Significant association was found between teachers´ level of education and knowledge of the SHP; teachers with university degree had better knowledge compared with teachers with Diploma and N.C.E;*
- *Most teachers do not know their roles in the SHP implementation needs; this shows that the teachers require training in the function of the SHP for effective programme delivery;*
- *Over 80% of the teachers have never seen the National School Health Policy document and over 90% of them have never been trained on the functioning of the SHP*.
